# Rugged Single Domain Antibody Detection Elements for *Bacillus anthracis* Spores and Vegetative Cells

**DOI:** 10.1371/journal.pone.0032801

**Published:** 2012-03-06

**Authors:** Scott A. Walper, George P. Anderson, P. Audrey Brozozog Lee, Richard H. Glaven, Jinny L. Liu, Rachel D. Bernstein, Dan Zabetakis, Linwood Johnson, Jill M. Czarnecki, Ellen R. Goldman

**Affiliations:** 1 Naval Research Laboratory, Washington D. C., United States of America; 2 Center for Bio/Molecular Science and Engineering, Naval Research Laboratory, Washington D. C., United States of America; 3 Nova Research Inc, Alexandria, Virginia, United States of America; 4 Biological Defense Research Directorate, Naval Medical Research Center, Silver Spring, Maryland, United States of America; Loyola University Medical Center, United States of America

## Abstract

Significant efforts to develop both laboratory and field-based detection assays for an array of potential biological threats started well before the anthrax attacks of 2001 and have continued with renewed urgency following. While numerous assays and methods have been explored that are suitable for laboratory utilization, detection in the field is often complicated by requirements for functionality in austere environments, where limited cold-chain facilities exist. In an effort to overcome these assay limitations for *Bacillus anthracis*, one of the most recognizable threats, a series of single domain antibodies (sdAbs) were isolated from a phage display library prepared from immunized llamas. Characterization of target specificity, affinity, and thermal stability was conducted for six sdAb families isolated from rounds of selection against the bacterial spore. The protein target for all six sdAb families was determined to be the S-layer protein EA1, which is present in both vegetative cells and bacterial spores. All of the sdAbs examined exhibited a high degree of specificity for the target bacterium and its spore, with affinities in the nanomolar range, and the ability to refold into functional antigen-binding molecules following several rounds of thermal denaturation and refolding. This research demonstrates the capabilities of these sdAbs and their potential for integration into current and developing assays and biosensors.

## Introduction

*Bacillus anthracis*, the etiological agent of anthrax, is capable of lethality in both animals and humans, and is a biothreat of great concern [1]. This is due in-part to its persistence and ease of production of the bacterial spore which can be disseminated as an aerosolized bioagent [2], [3], [4]. The threat posed by *Bacillus anthracis* is not new; research into its use as a bioweapon has been investigated since the early 1930s by a number of countries around the world. The accidental release of spores from a facility in the former Soviet Union which resulted in 42 cases of anthrax [3] and the Amerithrax incident of 2001 illustrate the devastating potential of this organism as a bioweapon.

The *Bacillus* genus is comprised of a diverse group of Gram-positive, aerobic bacteria, capable of forming an endospore. *Bacillus anthracis*, is a member of the *B. cereus* group which also contains the *B. cereus*, *B. thuringiensis*, *B. mycoides*, *B. pseudomycoides*, and *B. weihenstaphenesis* species. Within this group, the *B. anthracis*, *B. cereus*, and *B. thuringiensis* are highly similar morphologically and therefore difficult to differentiate using many laboratory techniques [5], [6], [7], [8]. The development of scientific tools such as PCR and RT-PCR have allowed for not only the differentiation of closely related *Bacillus* species, but also the identification of pathogenic versus non-pathogenic strains [9], [10], [11], [12], [13]. Additionally, novel and elegant techniques such as mass spectroscopic analysis of small acid soluble proteins [12], [14] and optical chromatography [15] continue to be developed. While these methods have all demonstrated their potential for success, each is limited in its ability to be incorporated into a biosensor that satisfies the requirements of ruggedness, portability, and simplicity.

Traditional antibodies have served as invaluable tools in medical and scientific assays for many years. Their high specificity and nanomolar binding affinities ensure that assays utilizing antibodies will not soon be replaced. However, while the traditional immunoglobulin is well-established, difficulties such as the cost of production and protein stability in austere environments can be encountered. To circumvent these complications, researchers have explored several recombinant forms including single chain Fv (scFv) antibodies derived from the variable regions of conventional antibodies [16], [17], and single domain antibodies (sdAbs) consisting of a single variable domain derived from heavy-chain only antibodies [18], [19], [20], [21]. The sdAb is derived from novel immunoglobulins found in sharks and members of the *Camelidae* family (camels, llamas, alpacas) that is comprised of only a heavy chain subunit, lacking the light chain found in the more common antibody structure [19], [22]. The isolated variable domain of these heavy-chain only antibodies is capable of folding to form the antigen-binding domain, exhibiting binding affinities equivalent to the progenitor antibody. Additionally, sdAbs possess the characteristic of thermal stability. This quality can manifest as an elevated melting temperature, in some instances as high as 85°C; or an ability to denature when exposed to temperature extremes and subsequently refold to an active molecule over several cycles [23], [24], [25]. This combination, high affinity and remarkable thermal stability, make sdAbs well suited for integration into field-based assays.

The EA1 protein, originally described by Ezzell *et al.* as the extractable antigen, has been shown to be a highly antigenic protein present in *Bacillus anthracis* spores and vegetative cells [26], [27], [28]. Both EA1 and Sap are proteins of the *B. anthracis* S-layer, a paracrystalline protein shell that encapsulates the vegetative cell [28], [29], [30], [31]. The proteins are expressed differentially during culture growth, with EA1 displacing the Sap S-layer in late stage exponential growth and stationary phase [32]. The EA1 protein has been identified in both vegetative cell samples and bacterial spore preparations [33], [34], [35]. Although it has been shown that EA1 is not a spore protein and can be removed from spore preparations [36], the high antigenicity and prevalence of the protein in typical spore preparations suggest that this S-layer protein would serve as a suitable target for assays designed to detect both vegetative cells and spore preparations.

In this study sdAbs were isolated using a phage display library prepared from immunized llamas. Rounds of selection on spore preparations produced unique clones that were shown to recognize the S-layer protein EA1. The binding kinetics and thermal stabilities of six of the isolated sdAbs were determined in order to demonstrate their potential for use in field deployable assays for the detection of *B. anthracis*.

## Results

**Figure 1:**
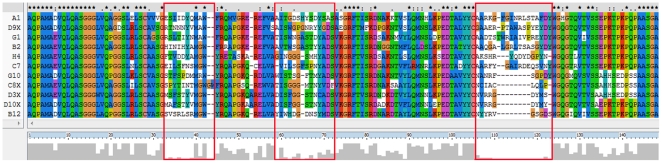
Amino acid alignments of sdAb families. Families were identified based on amino acid alignments and variability in the complementarity determining regions (CDRs) bracketed in red boxes. Single representatives of each family are shown here. Clones d3x, d9x, G1, H4, and C8x are members of families that are not described in this study.

The veterinary vaccine for anthrax, comprised of live *B. anthracis* Sterne 34F2 strain spores, was used for the immunization of two llamas. A library of sdAbs was constructed from the combined RNA extracted from the peripheral blood lymphocytes of both animals and was calculated to have a clonal diversity of approximately 5×10^6^ based on direct colony counts following electroporation and sequence analysis of a subset of individual clones. Of the 25 clones examined by sequencing, greater than 80% were dissimilar in amino acid composition of the complementarity determining regions (CDRs) and lacked any stop codons within the expected open reading frame. The library was subjected to two rounds of selection against *Bacillus anthracis* Sterne strain spores with the number of wash cycles increasing between successive rounds. Individual clones producing sdAbs with target affinity were identified using a monoclonal phage ELISA as described by Bradbury and Marks [37]. Sequencing and *in silico* analysis identified eleven families of sdAbs (Figure 1). Six of the families were chosen for further analysis. The other five families were either low affinity binders or determined to bind a similar epitope to one of the other sdAbs described in this work.

### Target determination

**Figure 2:**
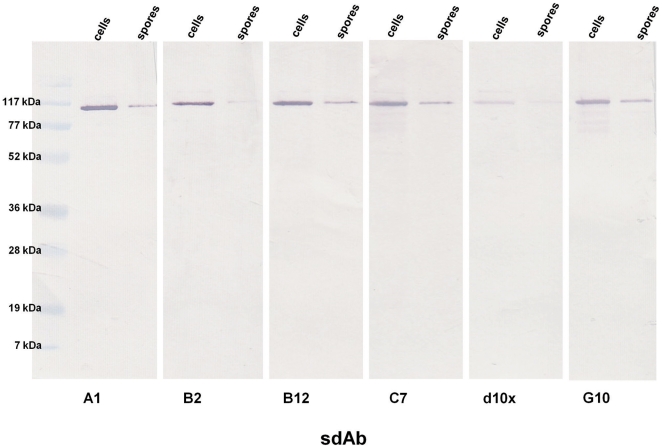
Immunoblot analysis of sdAb target protein. *B. anthracis* Sterne strain cell and spore lysates were separated via SDS-PAGE for immunoblotting using each of the sdAbs.

Despite the diversity of the CDRs between sdAb families, immunoblots of SDS-PAGE resolved vegetative cell lysates and disrupted bacterial spores showed that each of the purified sdAbs exhibited affinity for a single protein of approximately 100 kDa (Figure 2). Due to the size of the target and the fact that it was present in both vegetative and spore preparations, we hypothesized it was the S-layer protein EA1. Guanidinium soluble proteins were fractioned via FPLC and individual fractions were examined via SDS-PAGE and subsequent immunoblotting to identify those fractions containing the antigenic protein. The Gel Code Blue stained gel confirmed a high degree of purity of the target protein, (Figure S1). Mass spectroscopic analysis of the protein recognized by the sdAbs was generously provided by Dr. Dasha Leary and Dr. Judson Hervey of the Naval Research Laboratory (NRL). Mascot analysis of the spectroscopic data confirmed the protein as EA1 with greater than 65% coverage (data not shown).

### Specificity of isolated binders

**Figure 3:**
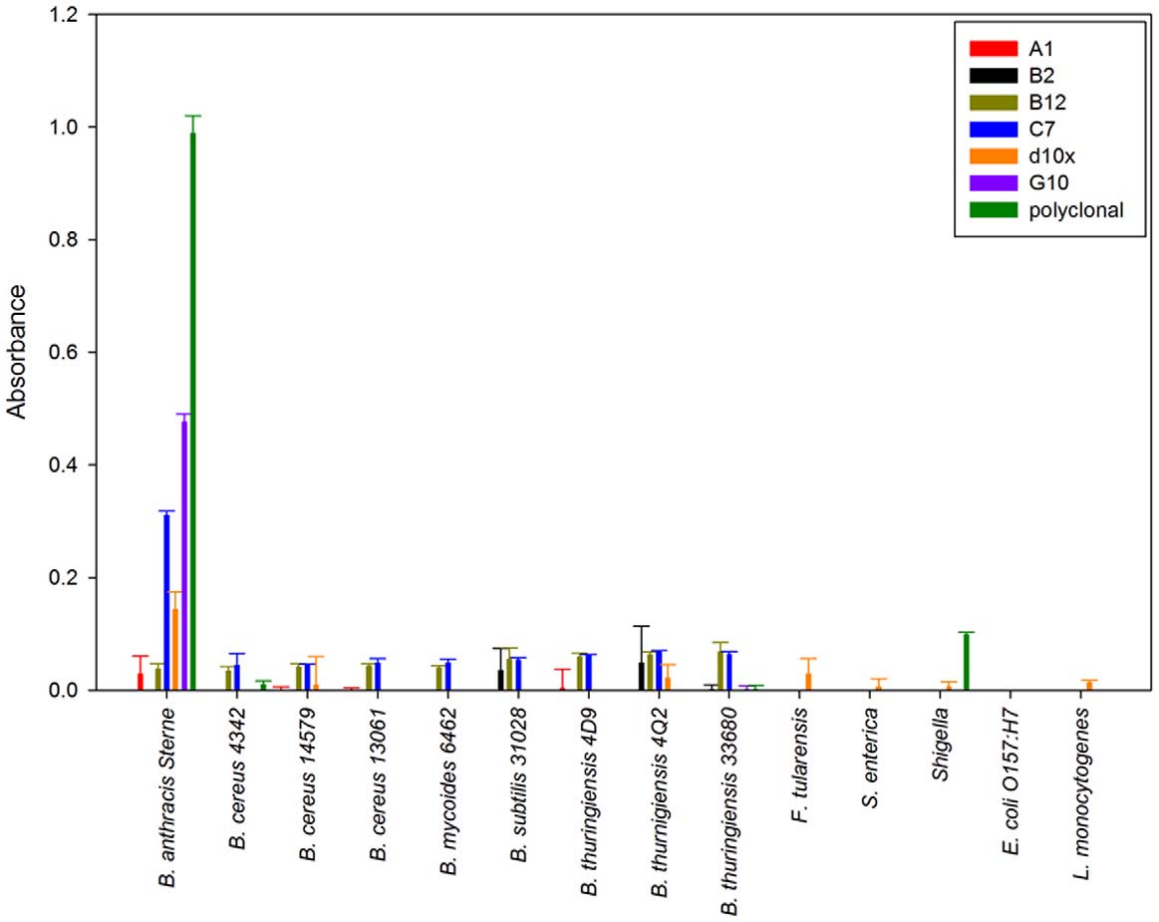
Specificity of sdAbs for whole bacteria. Bacteria were either grown overnight as live cultures or obtained from either CRP or KPL as killed material. Equivalent optical densities were immobilized to microtiter dishes for analysis of specificity using a direct binding ELISA.

**Figure 4:**
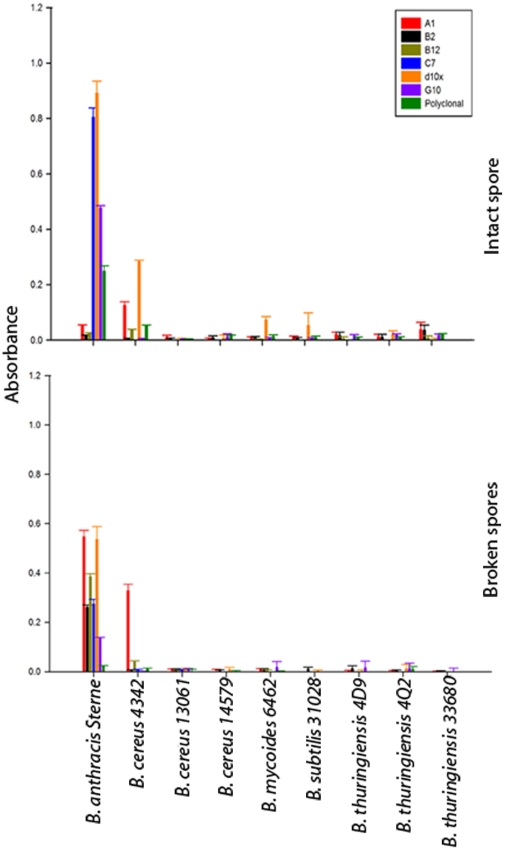
Specificity of sdAbs for *Bacillus* species spores. Intact and broken spores were passively immobilized to microtiter plates at equivalent optical densities to assess specificity of sdAbs.

To assess specificity of the isolated sdAb a direct binding ELISA with several representatives of the *B. cereus* group (*B. anthracis* Sterne 34F2, *B. cereus* ATCC 4342, *B. cereus* ATCC 14579, *B. cereus* ATCC 13061, *B. mycoides* ATCC 6462, *B. thuringiensis* 4D9, *B. thuringiensis* 4Q2, and *B. thuringiensis* ATCC 33680) and additional non-target bacteria (*B. subtilis* ATCC 31028, *E. coli* O157:H7, *L. monocytogenes*, *S. enterica* serovar Typhimurium, *F. tularensis*, and *Shigella*) was employed. Minimal interaction (absorbance <0.05) with the non-target *Bacillus* species was observed for all of the sdAbs examined and no cross-reactivity with the non-*Bacillus* bacteria in the panel (Figure 3). The sdAbs C7, d10x, and G10 demonstrated affinity for the vegetative cell; while the sdAbs A1, B2, and B12 bound the target only minimally. Similar results were observed when the bacterial spores were directly immobilized on microtiter plates. Given that all six sdAbs bound the EA1 protein in immunoblots, a second ELISA was performed in which the spores were first broken with a combination of boiling and sonication to fracture and release the exosporium and loosely attached proteins from the spore. The suspension, with no prior centrifugation, was then immobilized to the microtiter plate to allow for a mix of intact spores, exosporium fragments, and proteins on the well surface. In contrast to the results of the intact spore ELISA; the A1, B2, and B12 sdAbs showed a significant improvement in antigen binding suggesting an epitope of the EA1 protein that is not exposed in the intact spore (Figure 4).

**Figure 5:**
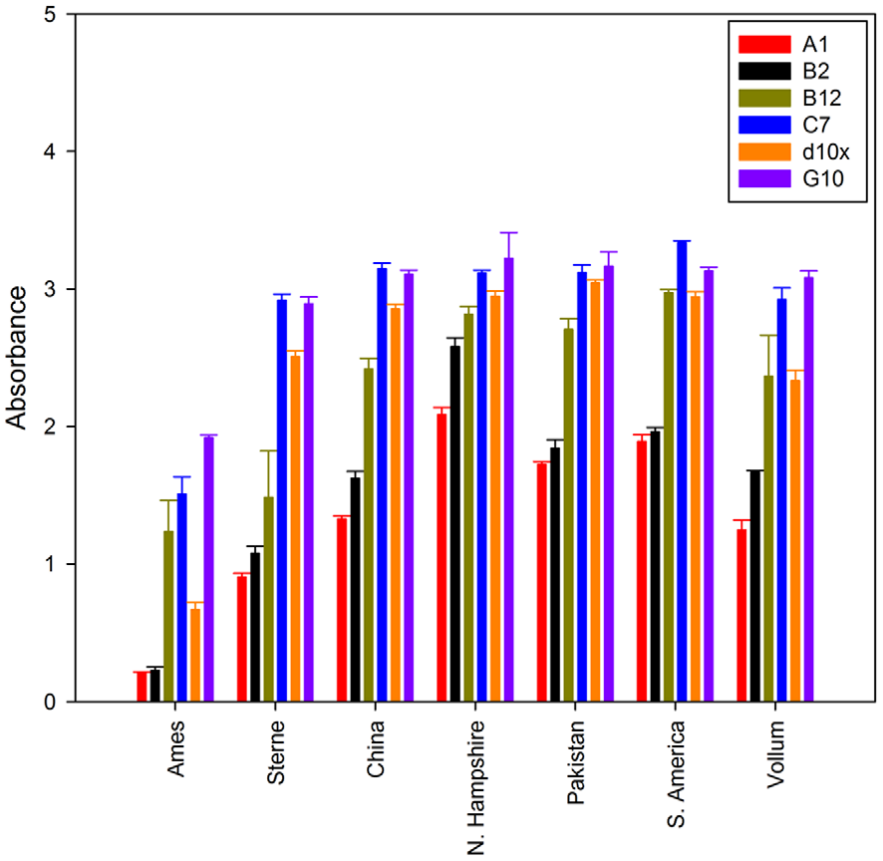
Affinity of sdAbs for *Bacillus anthracis* strains. Biotinylated sdAbs were tested for their ability to recognize a panel of *B. anthracis* strains from a number of geographic locations (acquired as irradiated spore preparation from NMRC).

Given the high degree of specificity for *B. anthracis* Sterne spores and cells, irradiated spores from *B. anthracis* strains from several geographic locations were acquired from the Navy Medical Research Center (NMRC) for further testing against pathogenic strains. The sdAbs were tested in a direct binding assay to assess their affinity for *B. anthracis* strains Pakistan, South America, China, Ames, N. Hampshire, Vollum, and Sterne (Figure 5). Each of the sdAbs bound the target spores comparable to previous assays against the Sterne strains spores. Those sdAbs hypothesized to bind an epitope not immediately accessible in intact spores (A1, B2, and B12) generally exhibited a lower affinity for the bacterial spores compared to the affinity of those thought to bind a more prominent surface feature (C7, d10x, and G10). It could not be determined if the variability in affinity shown by the sdAbs for the different spore preparations was the result of differences in the target epitope or simply the methods used to purify the spores themselves. Examination of the spores using phase contrast microscopy confirmed significant differences in the ratio of phase dark to phase bright spores as well as the percentage of intact spores compared to broken. Additionally, since the A1, B2, and B12 sdAbs showed substantial binding interactions with the immobilized spore preparations, it is likely the samples contained either exosporium fragments or free EA1 in addition to intact bacterial spores.

### Thermal stability of the isolated sdAb

**Figure 6:**
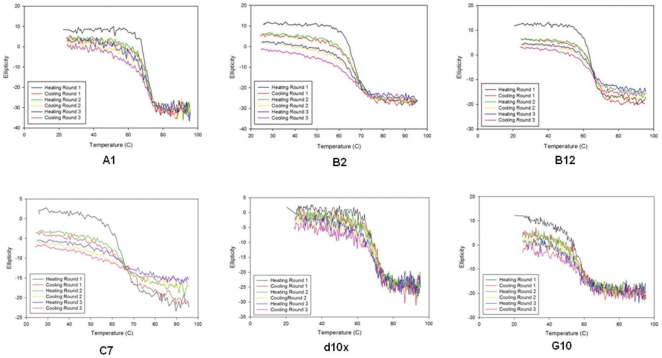
Determination of melting temperature and thermal stability of sdAbs. The ellipticity of polarized light at 205 nm was measured over a temperature range (25–95°C) to determine the melting temperature of each sdAb and its ability to refold folding thermal denaturation.

An inherent characteristic of the sdAb structure is its thermal stability. This refers not only to elevated melting temperatures, but also the ability of the structure to denature and subsequently refold into an active, antigen-binding molecule. Circular dichroism, which is frequently used to examine the secondary structure of proteins in solution, was employed to determine both the melting temperature of each of the sdAbs and to monitor the unfolding and refolding of the protein through several temperature cycles (25–95°C; Figure 6). Despite the high degree of conservation of the amino acids in the framework regions, each of the sdAbs tested melted at a unique temperature ranging from 57.9°C at the low end (G10) to 70.4°C (d10x). Similarly, each sdAb exhibited varying success in refolding to the native structure as assessed by monitoring the total change in ellipticity following one complete heating and cooling cycle (Table 1). Although this analysis suggested a significant loss of activity after the third round of thermal cycling, each of the sdAbs continued to show sufficient antigen binding activity when tested with both SPR and via ELISA, (Figure S2). When used at the same concentration employed in our typical ELISA (1 µg/ml) there was virtually no loss in the signal for any of the sdAbs except B2. This suggests that despite loss of some functional protein, sufficient active antibody remains to be successfully employed in our current assays.

**Table 1:** Melting temperatures and recovery of native structure.

	Melting Temperature (°C)	Round 1	Round 2	Round 3
A1	69.9	94%	86%	84%
B2	67.3	85%	73%	62%
B12	64.1	85%	73%	62%
C7	65.6	71%	48%	36%
d10x	70.4	93%	86%	79%
G10	57.9	78%	65%	58%

**The recovery of native structure (rounds 1–3) was calculated from the total change in left and right ellipticity for each cycle of heating and cooling compared to the initial total ellipticity.**

### Binding kinetics

The binding kinetics for each of the sdAbs was calculated following SPR analysis using the Bio-Rad ProteOn XPR36 system (Table 2). SdAbs were immobilized to a standard GLC chip to determine their binding kinetics for the purified EA1 protein. Binding affinities for the purified EA1 protein were in the nanomolar range for each of the sdAbs except d10x, although there was some variability as observed with the low association rate for B2 and the order of magnitude increase in dissociation rate for B12 compared to the other sdAbs tested. Despite several attempts, d10x failed to perform in SPR-based assays, possibly due to the immobilization strategy or the conditions of the surface regeneration step.

**Table 2:** Binding kinetics for sdAbs.

Clone	ka (1/Ms)	kd (1/s)	KD (M)^a^
A1	7.66E +5	2.33E −4	3.05E −10
B2	6.00E +4	3.70E −4	6.17E −9
B12	2.42E +5	1.50E −3	6.18E −9
C7	1.58E +5	2.48E −4	1.57E −9
d10x	NA	NA	NA
G10	8.11E +4	1.66E −4	2.04E −9

a KD values are calculated from kd/ka.

NA = No binding observed.

### Sandwich assays for the detection of *B. anthracis*

A multiplex Luminex bead-based assay was employed to assess the limit of detection in a sandwich assay. The sdAb monomers did not serve as suitable detection molecules in initial trials, recognizing only bacterial and spore concentrations of 10^6^ cfu/ml. The limit of detection, however, was significantly improved when sdAbs were tested as the dimeric alkaline phosphatase fusions [38]. The alkaline phosphatase fusions were able to reproducibly detect concentrations of 10^4^ cfu/ml, regardless of the combination of capture or tracer sdAb. It could not readily be determined which factor was responsible for the improved signal but possible contributors are improved immobilization of active molecules due to an increase in available amine-containing residues, increased affinity as a result of avidity, or simply a greater number of available biotin molecules on the larger dimer for signal amplification with the phycoerythrin – streptavidin reporter. Although not explored in this work, other fusion constructs have been examined and could potentially lead to alternate methods of immobilization or signal amplification that may further improve detection assays.

## Discussion

The objective of this project was the development of sdAbs toward *Bacillus anthracis* to provide recognition reagents with improved thermal stability. The sdAbs reported here were determined to be targeting epitopes on the S-layer protein EA1. Based on our results, it appears that there are at least two distinct epitopes recognized by the sdAbs examined in this study, (Figure S3). Three of the sdAbs clearly bound the vegetative cell and spore preparations, recognizing a readily accessible epitope of EA1 (C7, G10, and d10x). In contrast, the other sdAbs examined (A1, B2, and B12) were only able to bind the target antigen once freed from the bacterial spore suggesting an epitope buried within the exosporium of intact spores or the peptidoglycan layer in whole bacteria. The EA1 is a moderately large protein (862 amino acids) that forms a crystalline array around vegetative cells. Couture-Tosi [39] used electron microscopy and negative staining to map the features of the S-layer lattice, distinguishing between the inner and outer face topography. Based on their results, it is reasonable to believe our two sdAb clusters likely bind to epitopes present on only one side or the other, however without co-crystallization studies or epitope mapping it is impossible to state this definitively.

Although sdAbs that bind to spore or cellular surface targets would likely be the best candidates for a detection assay requiring little sample treatment, the sdAb A1 was shown to be the most amenable to surface attachment and displayed the highest affinity for the EA1 protein. With minimal sample treatment (boiling and/or sonication) this antibody may be best suited for integration into existing assays as a capture molecule.

In this study, both vegetative cells and spores were detected in ELISAs using each of the sdAbs described herein. The EA1 protein is not a required component of the spore, however it is commonly associated with the spore surface and can therefore serve as the targeted substrate for vegetative cells and bacterial spores, unless deliberate efforts have been undertaken to remove it from the spore preparations. Although there was a reduction in the signal with increasing number of washes (plateau reached at 5 washes), spores were consistently detected in ELISAs to concentrations of 10^6^ cfu/ml; regardless of the number of washes performed during spore preparation. More laborious spore preparation protocols can be employed to completely remove EA1, as has been shown in the literature [36], but would likely complicate the large-scale production of bacterial spores required for weaponization. Additionally, while the bacterial spore would be the primary target in the development of an assay or sensor for the purpose of biodefense, the ability to definitely identify *B. anthracis* in an unknown bacterial sample may prove advantageous in other diagnostic settings; eliminating the need for time consuming culturing or PCR based methods. An assay that utilized a combination of sdAbs, recognizing different targets on the bacterial surface, would ensure the greatest success in *B. anthracis* detection. The sdAbs developed in this research may work best paired with sdAbs targeting specific spore proteins to avoid the potential loss of detection capabilities by simply removing the EA1 protein from spore preparations.

*Bacillus* is a very diverse genus; while these sdAbs were found to be highly specific for the *B. anthracis* target, the assays conducted in this work focused on comparison to members of the *B. cereus* group. A simple BLAST search with the amino acid sequence for EA1 shows a range of identity scores ranging from 99 – 19% identity based on the species and the sequence stored at the National Center for Biotechnology Information. There is no protein sequence reported for the S-layer protein of several of the bacteria included in this study, so it is not possible to speculate if the observed specificity is strictly the result of substantial differences in the S-layer proteins themselves. Additionally, the strains with the highest identity scores were not represented in the panel of bacteria included in the specificity assays. It is feasible that the sdAbs would recognize S-layer proteins from other species that produce a S-layer protein more homologous to EA1. Future work should include strains with more similar S-layer proteins.

While sdAbs have yet to replace conventional antibodies as the recognition element of choice for most assays, recent publications suggest this to be an advancing field. As with other recombinant proteins, sdAbs can readily be produced in *Escherichia coli* or yeast expression cultures, significantly decreasing the cost of developing antibody-based assays and sensors. Additionally, as has been explored with scFv constructs, the small size of the gene and the bacterial host allows for a system to improve the characteristics of individual clones through mutagenesis, something not easily accomplished with mammalian or cell culture produced antibodies. Due to these advantages and their remarkable thermal stability these unique antigen-binding structures promise improvements that may enable testing at the point of concern.

## Materials and Methods

### Materials

Unless otherwise specified, chemical reagents were acquired from either Sigma Aldrich, Fisher Scientific, or VWR International. Restriction endonucleases and ligation reagents were from New England Biolabs. DNA amplification was accomplished with Roche Expand High Fidelity DNA polymerase kit. Specific laboratory kits and assays are defined where applicable.

### Library Construction and Selection

Two llamas (Triple J Farms, Bellingham, WA) were subjected to four rounds of immunization with the veterinary cattle vaccine for *anthrax* (Colorado Serum Company) that is comprised of live bacterial spores from the *B. anthracis* Sterne strain 34F2, an attenuated strain lacking one of the plasmids necessary for lethality. Institutional Animal Care and Use Committee (IACUC) approval for the immunization protocol was through the Triple J Farm protocol application process. To ensure the health and well-being of the animals, a modified vaccination dosage that utilized 1/5^th^ the injection volume typically used for cattle was employed, however multiple boosts were made to induce a robust immune response. Following immunization, peripheral blood lymphocytes were extracted from the buffy coat and RNA was isolated using the QIAamp RNA Blood Mini Kit (Qiagen) according to the manufacturer's protocol. Purified RNA was converted to cDNA using the SuperScript II Rt-PCR kit (Invitrogen). The sdAb library was constructed using primers specific for regions flanking the variable domain as outlined by Ghahroudi [40]. The variable domain genes were purified from successful reactions and cloned into the phage display vector, pECAN21 [41], via a T4DNA ligase ligation reaction with *SfiI*-digested vector and insert at a 3∶1 ratio. Ligation products were transformed into electrocompetent XL1-Blue cells (Strategene). Library diversity was calculated based on a direct count of colony forming units on output plates following ligation/transformation and sequence analysis of 25 clones to directly examine the sequence variability and ensure an open reading frame (ORF) in the majority of clones.

The library was subjected to several rounds of selection utilizing a modified protocol of that initially described by Griffiths *et al.* [42]. *Bacillus anthracis* Sterne strain spores were inactivated and fragmented using a combination of boiling and sonication as described below. The broken spores were diluted in PBS (pH 7.4) then passively immobilized to a microtiter plate to serve as the selection antigen. Following two rounds of selection, individual clones producing target-binding sdAbs were identified by monoclonal phage ELISA. Sequence analysis of purified plasmid DNA was performed by Eurofins MWG – Operon (www.operon.com). 

Single domain antibodies were grouped into families based on variations in the complementarity determining regions (CDRs) following alignment of amino acid sequences (ClustalW). Representative sdAb genes from each family were cloned into the periplasmic expression vector pECAN45 [43] and transformed into the Rosetta *E. coli* strain (Novagen). Expression and purification were performed as previously described [41], [44]. Following immobilized metal affinity chromatography (IMAC); sdAbs were purified from residual, contaminating proteins using a Superdex 75 10/300 GL column and Akta FPLC system (GE Healthcare). Quantitation of protein concentration was accomplished using either a NanoDrop1000 instrument and calculation based on the absorbance at 280 nm or a micro-BCA Assay (Pierce).

Each sdAb gene was also cloned into a modified pECAN45 vector that produces an alkaline phosphatase fusion with the sdAb protein [38]. The molecule dimerizes during expression to form a bivalent molecule. Purification of the dimer is accomplished using the same protocol employed with the monomeric form with the exception that a Superdex 200 10/300 GL column was used for FPLC purification.

### Bacterial Strains and Culturing

The various *Bacillus* species were obtained from either the American Type Culture Collection, *Bacillus* Genetic Stock Center (http://www.bgsc.org/), or NMRC; for a complete listing see Table S1. Other bacterial targets were obtained as a killed, bacterial antigen from the Critical Reagents Program (CRP), or KPL, Inc. Live *Bacillus* cultures were grown overnight at 30°C in either Tryptic Soy Broth or Luria Bertani for ELISA vegetative cell assays. Bacteria were pelleted at 3000× g then resuspended in PBS. The process was repeated three times to ensure residual medium was removed. The optical density at 600 nm (OD_600_) was measured using a Nanodrop 1000 instrument. For spore preparations, *Bacillus* species cultures were plated to the appropriate solid medium (Table S1, [45], [46], [47]) and incubated at 30°C for 3–4 days. Sporulation was assessed using an Olympus BX53 Phase contrast microscope system. Once greater than 95% sporulation (as determined by direct counts) had been obtained, cells were dislodged with 4°C sterile water and transferred to 2 ml microfuge tubes. Spores were pelleted at 10,000× g. Cellular debris and remaining intact cells were carefully removed as an upper layer, distinct of the spore layer. The centrifugation and removal of supernatant was repeated 5–10 times, until such time as the spore preparation appeared free of intact cells when examined via phase contrast microscopy.

Broken spores were prepared using a combination of boiling and sonication. Spores were diluted in PBS (pH 7.4) then boiled for 20 minutes. The spore suspension was placed on ice for 10 minutes then sonicated for 5 minutes alternating one minute sonication and one minute on ice using a Branson Sonifier (constant duty cycle, output control 5).

### Characterization of sdAb

Purified sdAbs were biotinylated to facilitate later ELISAs and immunoblotting experiments. The sdAbs were incubated at room temperature with a 10-fold excess of Biotin-LC-LC-NHS (Pierce) then passed through a Bio-Gel P-10 size exclusion column (BioRad) to remove excess biotin. An alkaline phosphatase-conjugated streptavidin (Prozyme) served as the secondary detection element in subsequent experiments.

Assays against target and non-target bacterium; including nearest neighbor *Bacillus* species, were conducted using a direct binding ELISA. Bacteria were immobilized at equivalent optical densities (OD_600_) to allow for qualitative analysis of data. Assays were performed as previously described, using 1 µg/ml of the biotinylated sdAb followed by a streptavidin- alkaline phosphatase fusion (Prozyme) [48]. Detection and measurement of sdAb binding was made possible using the colorimetric substrate SigmaFast *p*-Nitrophenol phosphate substrate (Sigma) following the manufacturer's protocol. The same direct binding ELISA protocol was followed for assays in which irradiated spores, spore preparation, and broken spores were immobilized. Sandwich ELISAs were also performed as described previously [48]. Briefly, capture sdAbs (1 µg/ml diluted in PBS) were immobilized on wells of a 96-well plate and dilutions of target applied prior to detection using biotinylated sdAb followed by the streptavidin- alkaline phosphatase fusion.

The target antigen was identified through immunoblot analysis. Late stage vegetative cells (16–20 hour) and purified spores were lysed/broken using a combination of boiling and sonication as previously described. The proteins of the soluble fractions were separated on a 14% SDS-PAGE gel then stained for visualization using a Gel Code Blue reagent (Pierce) or transferred to nitrocellulose for subsequent immunoblotting. Immunoblots were developed with the appropriate biotinylated sdAb (1 µg/ml in PBS containing 0.1% Tween-20 and 5% milk proteins) as the primary antibody and a streptavidin- alkaline phosphatase fusion (Prozyme) as the reporter. Visualization of binding interactions was accomplished with the Novex AP chromogenic substrate NBT/BCIP substrate (Invitrogen).

The melting temperature and renaturation capabilities of sdAbs were assessed via circular dichroism. Purified sdAbs were dialyzed overnight in 5 mM sodium borate solution (pH 7.5) then diluted to a final concentration of 30 µg/ml in the same solution prior to analysis using a Jasco J-815 CD spectrometer. The differential absorbance of the sample at 205 nm was measured over a temperature range of 25–95°C. The temperature was increased incrementally at a rate of 5°C/min. Melting temperature was defined as the inflection point between the folded and unfolded molecule. The percent recovery following heat denaturation and subsequent renaturation was determined by calculating the percentage of native protein ellipticity recovered.

Binding kinetics were calculated based on SPR measurements using a BioRad ProteOn XPR36 instrument. Purified sdAbs were diluted in 10 mM sodium acetate buffer (pH 4.0) to a concentration of 5 µg/ml then immobilized to a GLC chip (BioRad) using a standard EDC/NHS cross-linking reaction. Following immobilization of the target protein, remaining carboxyl groups were blocked with a 1 mM ethanolamine solution. Kinetic values (ka, kd, KD), were determined for each of the sdAbs by flowing six concentrations of purified EA1 over the surface at a flow rate of 50 µl/min for 3 min. Dissociation was monitored for 10 min, the surface then regenerated with a 50 mM glycine solution (pH 2.0). Calculations to determine binding kinetics were performed with the ProteOn Manager RM 2.1 software.

The limit of detection for bacterial spores was determined using a Luminex bead-based assay system. The monomeric sdAbs and alkaline phosphatase dimers of each sdAb were immobilized to commercial beads (Luminex) using the same carbodiimide reaction described for the SPR experiments. Intact *B. anthracis* Sterne strain bacteria and spores were diluted to concentrations of 10^6^ – 10^2^ cfu/ml in PBS buffer. Biotinylated proteins, monomers and alkaline phosphatase dimers of the sdAbs, were used as the detection element in sandwich style assays with a phycoerythrin – streptavidin conjugate serving as the tracer molecule.

### Purification of EA1

The S-layer protein (EA1) was enriched in vegetative cultures grown in SPY medium (60 mM K_2_HPO_4_, 45 mM KH_2_PO_4_, 15 mM (NH_4_)_2_SO_4_, 10 MgSO_4_, 2.4 mM sodium citrate, 0.2% (w/v) glucose, 0.2% (w/v) yeast extract) at 30°C overnight. The cells were pelleted at 3000× g for 15 minutes and the supernatant decanted. The cell pellet was washed twice in PBS then resuspended in 5 ml/g of cell material of 6 M guanidinium hydrochloride. The suspension was placed at 4°C on a rotisserie (Dynal Biotech) for 3 hours. Cell material was again pelleted at 3000× g and the supernatant dialyzed in 12–14000 MWCO tubing (SpectraPore) for 48 hours in PBS buffer with several buffer exchanges. The EA1 protein was separated from other soluble membrane proteins using the Akta FPLC system.

## Supporting Information

Figure S1 **Purification of the S-layer protein EA-1.** (A) Soluble and insoluble fractions of the cell lysate separated via SDS-PAGE for protein staining and immunoblotting (B) Chromatograph of FPLC fractionation of soluble fraction of cell lysate (C) Gel Code Blue stained SDS-PAGE analysis of individual fractions following FPLC.
(DOC)

Figure S2 **Binding activity of sdAb following thermal denaturation and renaturation.** Binding to immobilized EA1 following temperature cycling was assessed using a direct binding ELISA, SPR, and direct binding Luminex.
(DOC)

Figure S3 **Analysis of sdAb epitope.** Purified sdAbs were used in a sandwich ELISA, with purified EA1 as the antigen, to ascertain whether the sdAb recognized identical epitopes.
(DOC)

Table S1 **Bacterial strains and sporulation media.**
(DOC)
