# Supplementary material for: Rugged Single Domain Antibody Detection Elements for Bacillus anthracis Spores and Vegetative Cells
Source: PLoS One. 2012 Mar 6;7(3):e32801. doi: 10.1371/journal.pone.0032801 (PMC3295763; doi:10.1371/journal.pone.0032801)
Supplement: Figure S1 — Purification of the S-layer protein EA-1. (A) Soluble and insoluble fractions of the cell lysate separated via SDS-PAGE for protein staining and immunoblotting (B) Chromatograph of FPLC fractionation of soluble fraction of cell lysate (C) Gel Code Blue stained SDS-PAGE analysis of individual fractions following FPLC. (DOC) [file pone.0032801.s001.doc]

**Figure S1 – Purification of the S-layer protein EA-1**

S-layer proteins were enriched through growth in SPY medium. The cell pellet was incubated in 6M guanidinium hydrochloride to release soluble proteins the separated into insoluble and soluble fractions via centrifugation. The soluble and insoluble were resolved via SDS-PAGE. The left most image of Panel A is a GelCode Blue stained gel and the right an immunoblot using sdAb BA-G10. The soluble proteins were fractionated using an Akta FPLC system, Panel B, each of which was then examined via SDS-PAGE; Panel C. The fraction collected between 9 – 10 ml (fractions 6 and 7), highlighted by the blue box of Panel B, contained the target protein.

**Figure S1**

**
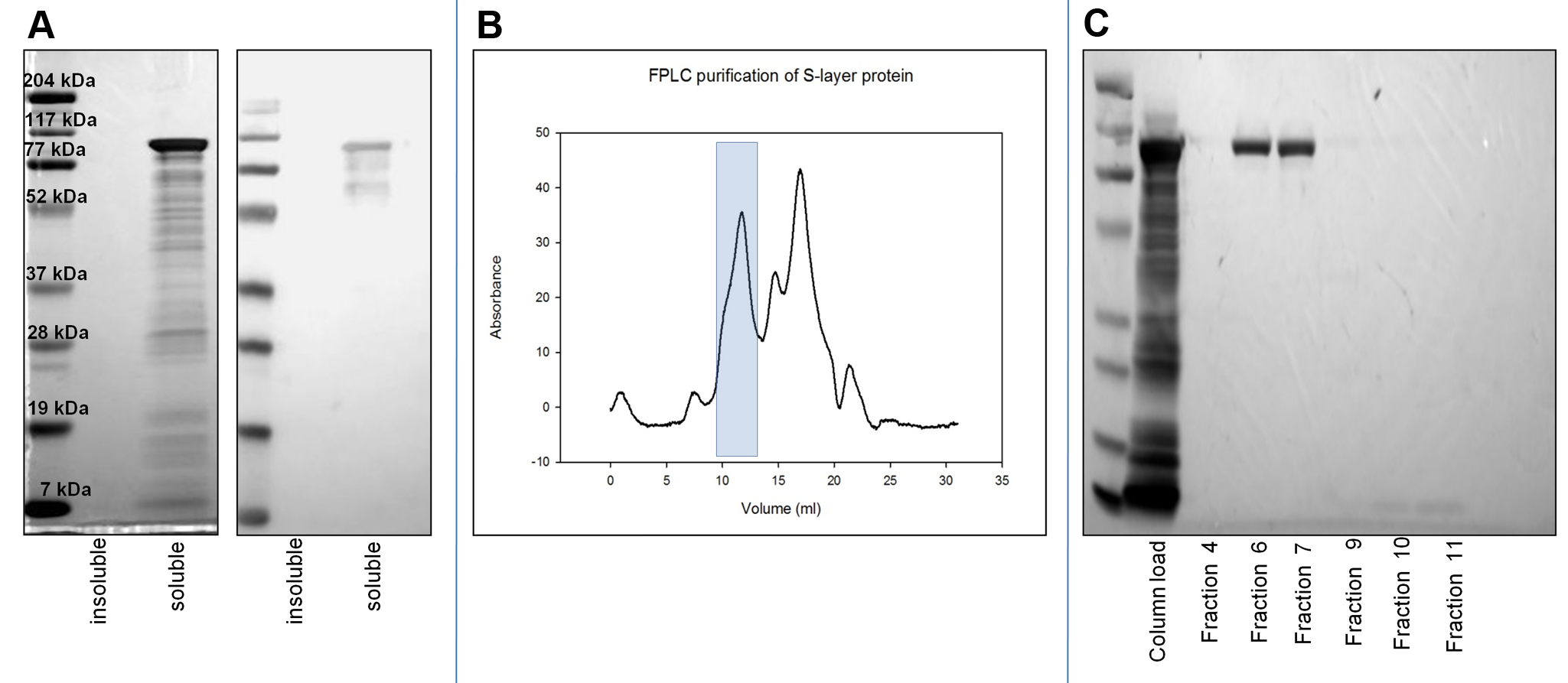
**
