# Supplementary material for: Rugged Single Domain Antibody Detection Elements for Bacillus anthracis Spores and Vegetative Cells
Source: PLoS One. 2012 Mar 6;7(3):e32801. doi: 10.1371/journal.pone.0032801 (PMC3295763; doi:10.1371/journal.pone.0032801)
Supplement: Figure S2 — Binding activity of sdAb following thermal denaturation and renaturation. Binding to immobilized EA1 following temperature cycling was assessed using a direct binding ELISA, SPR, and direct binding Luminex. (DOC) [file pone.0032801.s002.doc]

**Figure S2 – Binding activity of sdAb following thermal denaturation and renaturation**

The ability of individual sdAbs to bind the EA1 target was examined following rounds of heating (85-95 ˚C) and cooling (25 ˚C). Panel A. Aliquots were removed after each of three rounds of cycling (heating to 95 ˚C) and diluted to 1 µg/ml in blocking buffer for the ELISA. Each of the sdAb samples maintained a sufficient quantity of active sdAb to produce a measureable signal. In contrast, the control immunoglobulin sample demonstrated a complete loss of binding activity after the first round of heating. The B2 sample showed loss of activity with each successive round which coincides with the observations of the CD experiment. In contrast, the G10 sample which showed a gradual loss of activity in the CD experiments, continued to bind the target antigen with equivalent affinity. The affinity recovered (y-axis) is based on the ratio of the absorbance of the sample for each cycle. Panel B. Binding activity of the sdAbs was measured by SPR following heating to 95 °C. The SPR signal (resonance units) generated by the addition of 30 nM sdAb to a surface coated with EA1 was measured following the indicated number of heating cycles. Data is plotted as the percent signal relative to the unheated sdAbs. As with the ELISA, B2 shows a greater loss of activity on heat cycling than the other sdAbs. Panel C. Luminex bead-based direct binding assays were used to monitor the activity of two purified sdAbs and Llama IgG by evaluating a dose response curve, following thermal cycling (heating to 85 °C). Beads were coated with *B. anthracis* spore proteins (after sonicating Sterne strain spore preparations). Biotinylated sdAb and llama polyclonal antibody were added at the indicated concentration, and signal measured after the addition of phycoerythrin – streptavidin conjugate.

C
