# Supplementary material for: Rugged Single Domain Antibody Detection Elements for Bacillus anthracis Spores and Vegetative Cells
Source: PLoS One. 2012 Mar 6;7(3):e32801. doi: 10.1371/journal.pone.0032801 (PMC3295763; doi:10.1371/journal.pone.0032801)
Supplement: Figure S3 — Analysis of sdAb epitope. Purified sdAbs were used in a sandwich ELISA, with purified EA1 as the antigen, to ascertain whether the sdAb recognized identical epitopes. (DOC) [file pone.0032801.s003.doc]

**Figure S3 – Analysis of sdAb epitope**

Purified sdAbs were used in a sandwich ELISA, with purified EA1 as the antigen, to ascertain whether the sdAb recognized identical epitopes. The results of the assay are not conclusive as some of the sdAb are not efficiently immobilized as an active, antigen-binding molecule. The sdAb A1, however, which is known to passively immobilize in an active conformation (data not shown) suggests that a different epitope of EA1 is targeted by sdAbs C7, G10, and B12 than the A1 sdAb. It cannot be determined whether the other sdAbs bind the same epitope with different affinities (combined with the results of Figure 3) or epitopes that are proximal resulting in steric inhibition of binding.

**
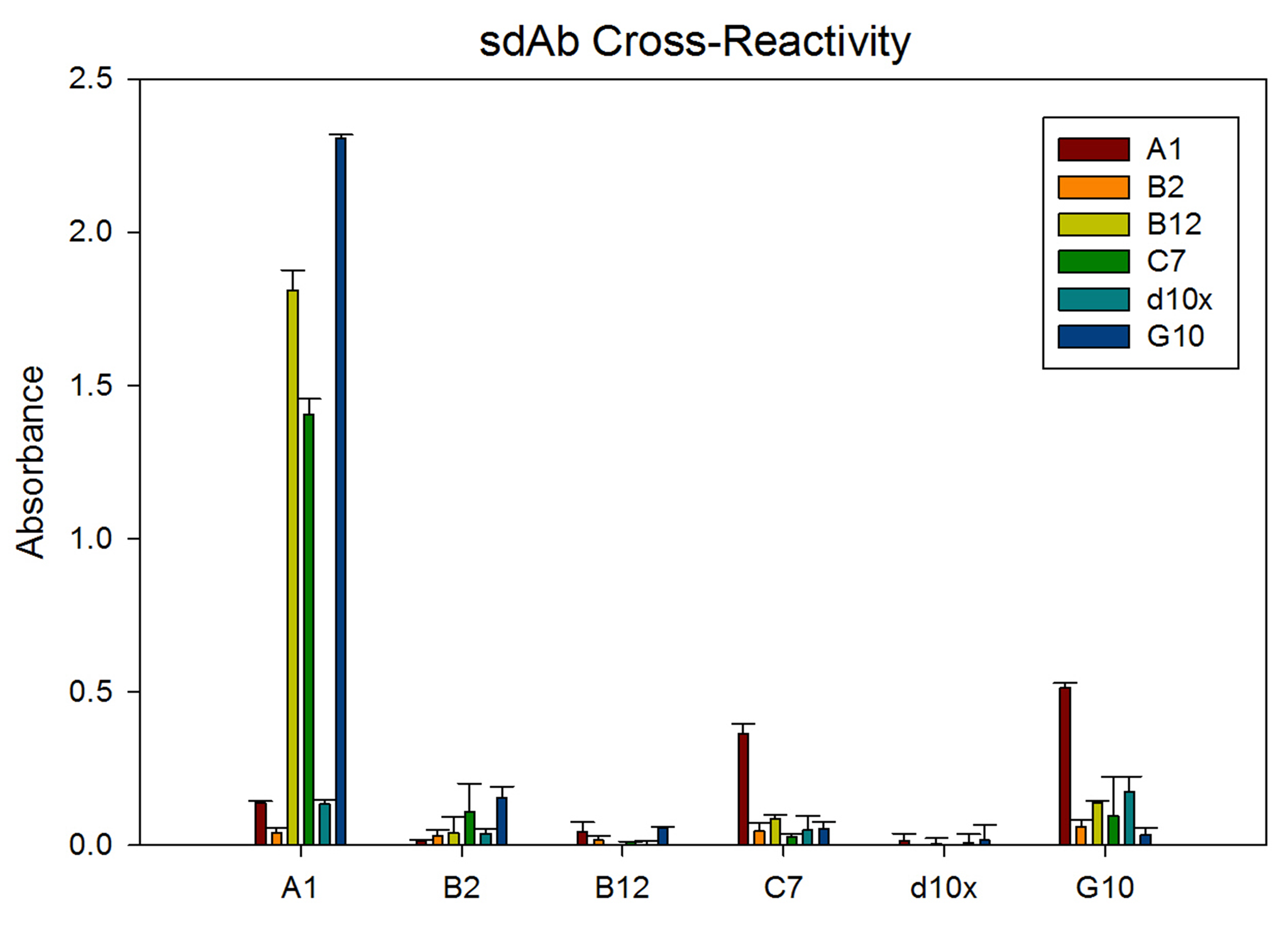
**
