# Supplementary material for: Rugged Single Domain Antibody Detection Elements for Bacillus anthracis Spores and Vegetative Cells
Source: PLoS One. 2012 Mar 6;7(3):e32801. doi: 10.1371/journal.pone.0032801 (PMC3295763; doi:10.1371/journal.pone.0032801)
Supplement: Table S1 — Bacterial strains and sporulation media. (DOC) [file pone.0032801.s004.doc]

**Table S1 – Bacterial strains and sporulation media**

| ***Bacillus* species** | **Sporulation Medium** |
| --- | --- |
| *B. anthracis* Sterne 34F2 * | 2xSG [46] |
| *B. anthracis* Ames, Vollum, Pakistan, South America, China, New Hampshire** | Did not sporulate |
| *B. cereus* ATCC 4342 *** | 2xSG [46] |
| *B. cereus* ATCC 14579 * | 2xSG [46] |
| *B. cereus* ATCC 13061 * | 2xSG [46] |
| *B. mycoides* ATCC 6462 *** | Schäffer [45] |
| *B. subtilis* ATCC 31028 * | Schäffer [45] |
| *B. thuringiensis BGSC 4D9 ** | NSM [44] |
| *B. thuringiensis BGSC 4Q2** | NSM [44] |
| *B. thuringiensis ATCC 33680** | NSM [44] |

* Bacteria were obtained from either the American Type Culture Collection (ATCC) or the Bacillus Genetic Stock Center. Generally, *bacillus* species were maintained on tryptic soy agar or broth until plated to the appropriate media for sporulation.

****** The pathogenic Ames and Vollum strains as well as the geographic *B. anthracis* samples Pakistan, South America, China, and New Hampshire were obtained as irradiated spores from the Navy Medical Research Center. These samples were not maintained as live cultures.
